# Development and Study of Sustainable Edible Coating from Carrageenan/Starch/Nanocellulose for Enhancing Fruit/Vegetable Shelf Life and Preservation

**DOI:** 10.1021/acsomega.5c00480

**Published:** 2025-03-27

**Authors:** Mariia Dmitrenko, Daniel Pasquini, Anna Kuzminova, Ilnur Dzhakashov, Sabu Thomas, Anastasia Penkova

**Affiliations:** † St. Petersburg State University, 7/9 Universitetskaya nab., St. Petersburg 199034, Russia; ‡ Instituto de Química, Universidade Federal de Uberlândia, Campus Santa Mônica, Av. João Naves de Ávila, 2121, Uberlândia, Minas Gerais 38400-902, Brazil; § International and Inter University Centre for Nanoscience and Nanotechnology, Mahatma Gandhi University, Kottayam, Kerala 686560, India

## Abstract

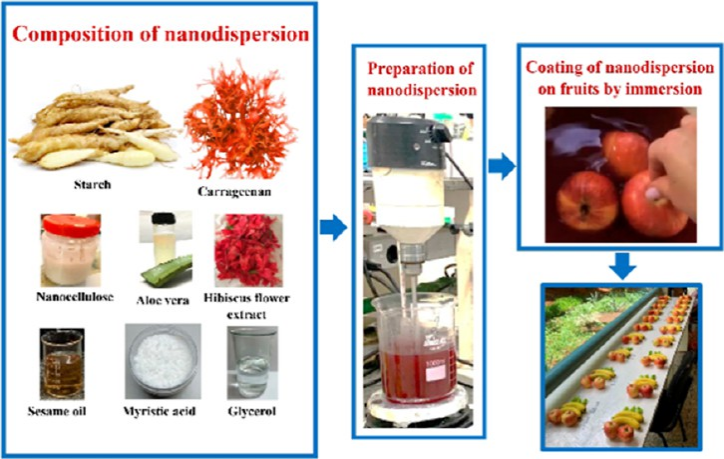

The packaging material must be safe for food, humans, and the environment, which makes the work on creating edible biodegradable packaging from polymers relevant. In this work, sustainable edible carrageenan/starch nanodispersions reinforced with nanocellulose (NC) for packaging (coating) of products were developed to improve their shelf life and preservation. The effect of the polysaccharide ratio and NC particle forms on nanodispersion properties and coating process was investigated. Various analysis methods were applied to study nanodispersions, determining particle shape, size, density, surface tension, viscosity, and contact angles onto fruits/vegetables. Nanodispersions were coated onto apples, bananas, and peppers for evaluation of their storage. The nanodispersions with 33.3/66.7 wt % carrageenan/starch with 5% NC fibrils or 10% NC crystals demonstrated the potential for applying on fruits as packaging due to decreased water loss from fruits/vegetables. They can be used prospectively by spraying on fruits/vegetables during harvesting since they consist of components actively used in the food industry.

## 1 Introduction

The shelf life of products can be reduced due to various factors such as respiration, microbial spoilage, etc.,^1^ which leads to a shorter shelf life, difficult transportation, and threat to human health. It is also worth noting that losses in agriculture due to spoilage of products are more than 40%.^2^ The use of appropriate strategies such as special coating/packaging from polymers, especially edible and biodegradable in the realities of the world, where there is active development and protection of the environment, is very relevant.

Synthetic polymers in the packaging field have negative impacts on the environment. Nonbiodegradable accumulation leads to global climate change and the gradual depletion of fossil reserves.^3^ Thus, the direction of sustainable development, which promotes environmental conservation, has led to active research into biobased polymers and the development of edible and biodegradable packaging materials. Many natural polymers such as cellulose, starch, alginate, chitosan, collagen, etc. have been used to develop potential product packaging.^4−6^ Edible packaging from biopolymers may be developed in two forms as a preformed film for product wrapping and as an edible coating (solution or dispersion) applied directly to the product for improving shelf life and postharvest quality of products.^7^ This work is a continuation of the development: previously, films were developed for use as potential packaging material^8^ while in this work, the option of using starch/carrageenan/nanocellulose (NC) dispersions as coatings for fruits/vegetables was considered.

The choice of the starch/carrageenan matrix was due to their unique properties and the possibility of their miscibility and compatibility in a wide concentration range,^4,9^ which made it possible to vary the properties of the dispersion obtaining the tailored ones for application to fruits. The advent of nanotechnology has led to intense interest and application in NC (<100 nm size), which is renewable, biocompatible, and biodegradable and is at the forefront of research.^10^ The addition of even small concentrations of NC in materials enhances mechanical properties.^11^ It is also in high demand in biomedical, paper, food, cosmetics fields, etc.^12−14^ However, it is really necessary to determine the influence of the two main geometrical NC forms (crystals (CNC) and fibrils (CNF)) and their critical concentrations, and there is not always a positive effect on changes in the properties of the material. It is also necessary to add plasticizers (low-molecular-weight agents), compatibilizers, surfactants, and additives for the preparation of edible films and coatings in order to improve properties or impart other functional properties.

Thus, based on the already obtained results for films, the aim of this study was to develop and study sustainable edible carrageenan/starch/NC nanodispersions for coating onto fruits/vegetables and to improve their shelf life and preservation. The novelty was the investigation of the effect of polysaccharide ratio and NC particle forms (CNC and CNF) in the wide concentration range on nanodispersion properties and the coating process. The incorporation of additional components such as aloe vera gel, glycerol, sesame oil, and hibiscus flower extract into this system was carried out to improve antioxidant, mechanical properties, flexibility, moisture resistance, and antibacterial properties. The antibacterial and antioxidant properties of aloe vera, hibiscus extract, and sesame oil are already well established in the literature.^15−17^ They are widely used worldwide as ideal natural food additives, and their industrial production is well established. The developed nanodispersions were studied by various analysis methods: dynamic light scattering method, density, surface tension, viscosity, and contact angle measurements to evaluate the prospects of using them as a packaging material for coating on fruits/vegetables such as apples, bananas, and peppers. The choice of these fruits/vegetables was because they had different acidity or alkalinity with short shelf life; they are available and actively used by human.

## 2 Experimental Section

### 2.1 Materials

Carrageenan and arrowroot starch (Kottayam, Kerala, India) were used as the matrix for nanodispersion preparation. NC (CNF with a length of a few hundred nm and a diameter of 20–10 nm, CNC with 215 ± 52 nm length and 4.9 ± 1.5 nm diameter, both extracted from Eucalyptus bleached Kraft pulp, Uberlandia, Brazil), aloe vera gel (Cathedral Pharmaceutical Industry, Brazil), and hibiscus flower extract (Uberlandia, Brazil) were used as reinforcing agents to enhance mechanical properties, to form film on products after deposition of nanodispersion, and to provide antibacterial and functional (color change depending on pH) properties, respectively. The detailed synthesis and characterization of reagents (polysaccharides, NC, hibiscus flower extract) were previously published.^8,18^ Sesame oil obtained from Pazze Food industry (Brazil) was used as a compatibilizer to improve hygroscopic properties,^8^ while myristic acid (≥99%, CAS 544-63-8, Sigma-Aldrich, Malaysia) was applied as a surfactant for the oil mixing with water.^19,20^ Glycerol from Dinâmica Company (Brazil) was added as a plasticizer to improve elastic properties.^8^

### 2.2 Preparation of Nanodispersions

Variation of the concentration of polysaccharides (carrageenan, starch, and NC) was carried out for the nanodispersion preparation, maintaining the concentration of other components: 0.3 wt % glycerol, 0.05 wt % sesame oil, 0.05 wt % myristic acid, 0.05 wt % aloe vera gel, and 0.5 wt % hibiscus flower extract in the nanodispersion. The ratio of carrageenan/starch was varied as 26.5, 33.3, 50, 66.6, and 73.5 wt % of carrageenan in the blend. The dispersion was prepared as follows: 1 g of carrageenan/starch with glycerol was dissolved in 100 mL of water at 80 °C with constant stirring until it reached a homogeneous state. The following components were then added in sequence: myristic acid, sesame oil, and aloe vera gel. After this, the dispersion temperature was reduced to 60 °C for adding hibiscus flower extract. The temperature reduction was due to obtaining a saturated color of the dispersion. NC was added last followed by intense stirring for 1 h. The NC content in the dispersion was varied.

The factorial design matrix for the dispersion development is shown in Table 1. To optimize the dispersion composition, variations were made in NC particle structure and content and the carrageenan/starch ratio as outlined in the factorial design in Table 2.

**Table 1:** Planning Matrix for Factorial Design Used to Develop Films

experiment	carrageenan, wt %	NC, wt %
0 (×2)	0	0
1	–1	–1
2	+1	+1
3	–1	+1
4	+1	–1
5	0	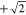
6	0	–
7	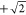	0
8	–	0

**Table 2:** Nanodispersion Designations and Compositions

levels	carrageenan content, wt %	NC content, wt %	
		CNF	CNC
(0;0)	50	10	7
(−1;–1)	33.3	5	4
(1;1)	66.6	15	10
(−1;1)	33.3	15	10
(1;–1)	66.6	5	4
(0; )	50	17.07	11.23
(0;)	50	2.92	2.77
(;0)	73.5	10	7
(;0)	26.5	10	7

In statistical analyses, treatments were coded to represent the levels of tested variables, typically as “high” and “low” or “absent” and “present,” denoted as “+” and “–” or “0” and “1.” The codes represent real values of the variables/factors,^21^ as detailed in Table 1. All statistical treatments used coded level values (−1, 0, +1, etc.), and equations were generated in a coded form. Concentrations of polysaccharides (carrageenan, starch, and NC) were varied while keeping other components constant. Carrageenan and starch levels were selected to encompass a full range of compositions (from 26.5 to 73.5 wt %). The constant contents of other reagents (sesame oil, myristic acid, aloe vera gel, and glycerol) were chosen based on literature review and previously obtained data for food packaging in a film form^8^ to ensure desired properties. The content of hibiscus extract was optimized in previous studies, with 0.5 wt % in nanodispersion being the threshold value for effective coloration, while higher amounts resulted in loss of transparency. A defined range of NC values was used to study film behavior around the percolation threshold.^22^ The NC concentration range varied between 2.77 and 11.23 wt % for CNC and 2.92 and 17.07 wt % for CNF, attributed to differences in structure and crystallinity. CNF possess both crystalline and amorphous phases, enabling a higher concentration in the polymer matrix than CNC.^23^ To ensure reproducibility and validate the statistical results, experiments at the central point (0,0) were conducted twice (×2 in Table 1).

### 2.3 Nanodispersion Characterization

#### 2.3.1 Dynamic Light Scattering Method

The determination of particle shape and size in nanodispersions was carried out using a Photocor Complex dynamic light scattering spectrometer (LLC Photocor, Russia). The original samples were diluted 20× with water. In the obtained dispersions, large particles and their aggregates were visible to the naked eye. The Photocor Complex instrument (Photocor, Russia) is designed for measuring sizes in the range from 0.5 nm to 5–6 μm. The majority of particles and their aggregates in nanodispersions were significantly larger; therefore, direct measurements were not conducted. Therefore, the samples were left to settle overnight for large scatterers to settle, and measurements were carried out in the supernatant part of the colloids. Measurements were performed at a wavelength of 654 nm at a scattering angle of 90° at 25 °C. The signal accumulation time was 300 s. Three consecutive measurements were conducted for each sample to check the reproducibility and assess the data variability.

#### 2.3.2 Viscosity Measurement

For the measurement of the dynamic viscosity of the nanodispersions using the rotational method of analysis (obtaining dependencies of shear stress and viscosity on shear rate), the following equipment was used: MCR 702 TDR rheometer (Anton Paar, Austria), coaxial cylinders CC-10 (radius 5 mm, minimum sample volume 1.2 mL), temperature control system C-PTD 200 (providing temperature control from −20 to 200 °C using Peltier elements for cylindrical systems), temperature set at 25 °C, temperature stabilization time of 300 s, and shear rates ranging from 1 to 1000 1/s (21 data points with a logarithmic step). The dependencies of shear stress on shear rate were approximated using Newton’s model (linear law) and a power law. Three consecutive measurements of the flow curves were performed for each sample. The shear rate was automatically set, the rotational torque was recorded, the shear stress was determined, and the dynamic viscosity was calculated.

The kinematic viscosity of nanodispersion samples was measured by a VISCO BASIC Plus Rotation viscometer in auto test configuration at 25 °C.

#### 2.3.3 Density Measurement

The density of nanodispersion samples was measured by a DMA 5000 M CK (Anton Paar, Austria) density meter at 20 °C.

#### 2.3.4 Surface Tension Measurement

The surface tension of nanodispersions were measured by the Du Noüy ring method using a K6 force tensiometer (KRÜSS) at 25 °C temperature control of the samples. This method measures the force exerted on the optimally wetted ring due to the tension of the withdrawn liquid plate when the ring is removed.

#### 2.3.5 Contact Angle Measurements

**Figure 1:**
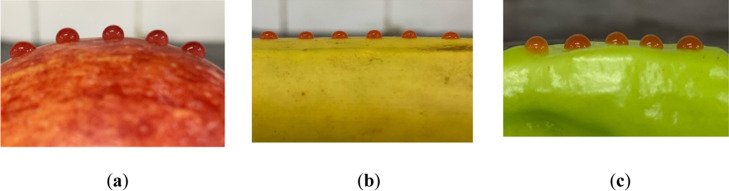
Contact angle measurements on (a) apples, (b) bananas, and (c) sweet peppers.

Contact angle for nanodispersions deposited onto fruits/vegetables (apples, bananas, and peppers) was measured by the “sessile drop” method (Figure 1) using the Goniometer LK-1 device (NPK Open Science Ltd., Krasnogorsk, Russia) and the “DropShape” program for analysis of the collected data. At least 9 measurements were made on each sample. The averaged values were used.

#### 2.3.6 Fruits/Vegetables Assessment after Nanodispersion Deposition

**Figure 2:**
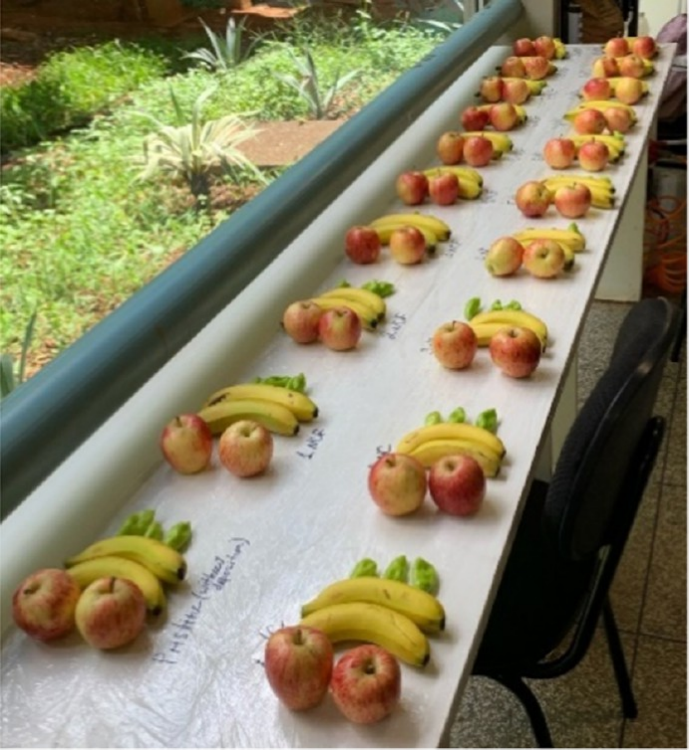
Storage and measurement of fruits/vegetables with/without deposited nanodispersions to measure the process of weight loss.

The apples, bananas, and sweet peppers were purchased from the same lot of a greengrocer (Uberlandia, Brazil). Before processing, the fruits/vegetables were individually visually inspected for uniformity of size and absence of defects, then rinsed with tap water, and dried overnight.^24^ Control and coated samples were randomly assigned. The samples were immersed in the nanodispersion for one min, then removed from the solution, and excess coating was removed by draining for at least 30 min, and dried overnight. The coated samples were stored at 27 °C (Figure 2). All experiments were performed in triplicate. The average values of three replicates with standard deviations (error bars) were used for the presentation. The weight of fruits and vegetables was measured by monitoring the weight changes during the storage period,^25^ and the weight loss was calculated using eq 1
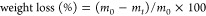
1where *m*_0_ is the initial weight of fruits and vegetables, and *m*_*t*_ is the weight of fruits and vegetables measured on *t* period.

#### 2.3.7 Scanning Electron Microscopy

Cross-section morphology of the films prepared from nanodispersions with optimal compositions ((−1;–1) composition with CNF and (−1;1) composition with CNC) was studied by using a TESCAN VEGA microscope of the model VEGA 3 LMU. The film samples were subjected to fracture after immersion in liquid nitrogen.

#### 2.3.8 Climatic Tests

Climatic tests were carried out for films prepared from nanodispersions with optimal compositions ((−1;–1) composition with CNF and (−1;1) composition with CNC) by using a heat-cold KTX-74-65/165 chamber (Smolensk SKTB SPU, Smolensk, Russia). The tests were carried out according to GOST 9.707-81, method 3 (https://plastinfo.ru/content/file/gosts/88f51fd741f6.pdf). The essence of the method consists of simultaneous conducting of accelerated tests of the material under study for resistance to the effects of climatic factors and the establishment of a comparative assessment of the resistance of materials to the specified effects based on the change in one or several characteristic aging indicators. For the tests, samples measuring 2 cm × 2 cm were cut out. The tests were carried out for at least 5 samples of each composition. According to GOST, the time for conducting cyclic climatic tests was calculated, which amounted to 20 cycles with a holding time of 12 h (1 cycle is equal to 12 h, where the first 6 h are held at 60 °C and the following 6 at −60 °C), which corresponds to 5 years of guaranteed shelf life. Humidity was not taken into account during the test. After the test, changes in the appearance of the samples (the occurrence of cracks, bubbles, and changes in geometric characteristics) were recorded.

#### 2.3.9 Film Light Transmission

Optical density (*D*) and light transmittance (*T*) of films prepared from nanodispersions with optimal properties were determined using a PE-5400UV spectrophotometer.^26^ The light barrier properties of a film sample of 10 × 45 mm were assessed using a glass cuvette, with air serving as the control, across wavelengths ranging from 350 to 750 nm.

#### 2.3.10 Solubility in Water

The water solubility (Sw) of the films prepared from nanodispersions with optimal properties was determined as follows:^27^ samples (2 × 2 cm) dried at 60 °C for 24 h to constant weight (*w*_i_) were immersed in vials with 15 mL of distilled water and left for 1 h at ambient temperature. The films were dried at 60 °C for 24 h and weighed (*w*_f_). Sw was calculated using eq 2
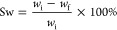
2

#### 2.3.11 Statistical Analysis

The results are presented as the mean ± standard deviation (SD) based on the results from at least three parallel measurements. Normal distribution of random variables was accepted, and outliers were excluded. The analysis of results, its random error, total unexcluded systematic error, and analysis of results total error were calculated.

## 3 Results and Discussion

### 3.1 Structure and Properties Investigation

**Figure 3:**
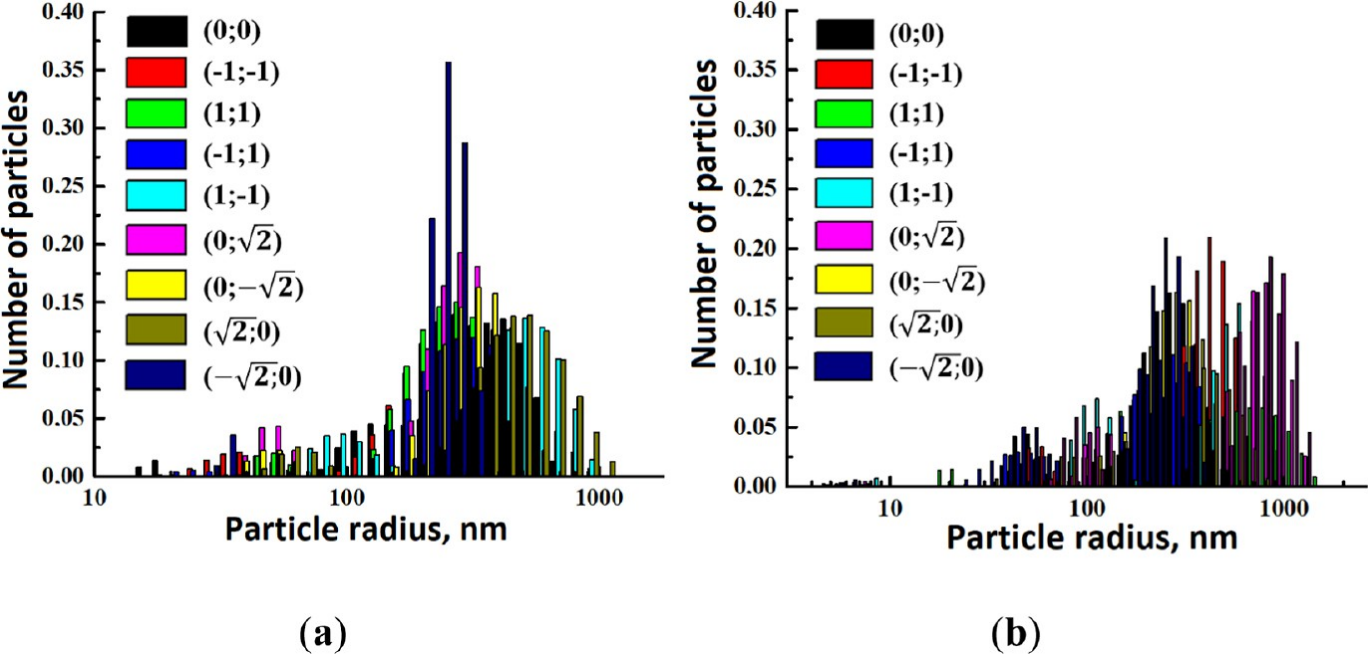
Dependence of the particle number on their radius for nanodispersions containing (a) CNF and (b) CNC.

The nanodispersion samples were characterized including the particle size distribution, viscosity, density, and surface tension. The particle shape and size are presented in Figure 3.

**Figure 4:**
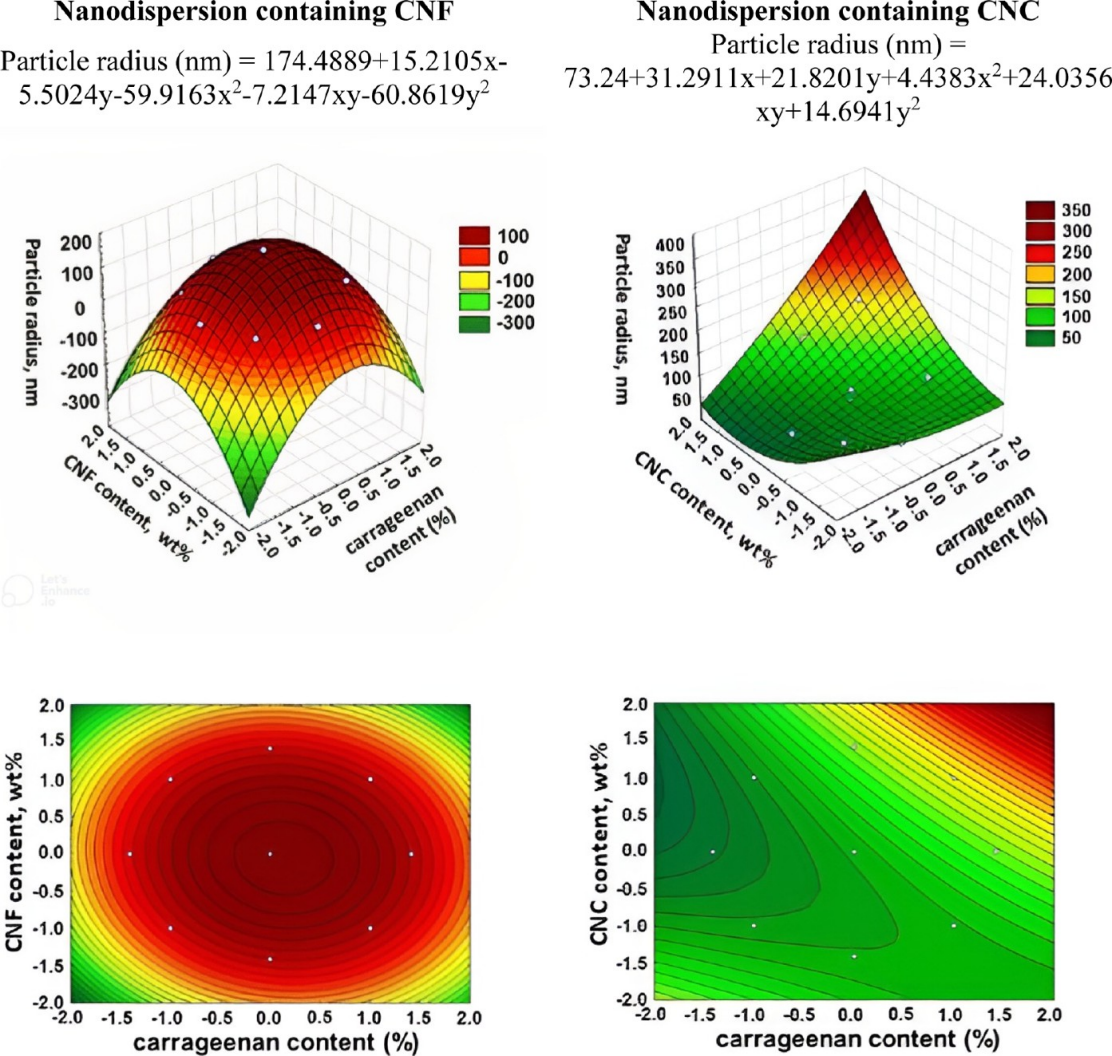
3D surface and contour plot graphs for the dependence of the particle radius of nanodispersions.

It was demonstrated that practically all obtained distributions were bimodal with average radii of R2 and R3. For some samples, particles of smaller radius R1 were visible but their contribution was very small, almost invisible against the background of large scatterers. It is important to consider analyzing the data to determine that the main part of particles and their aggregates settle at the bottom and are not reflected in the distributions. In fact, it can be considered that colloids contain particles with an average radius of R2 and particles with radii starting from R3 and larger. Based on the data presented in Figure 3, the average particle size R2 was selected, and obtained data from the statistical treatments are presented in Figure 4.

For nanodispersions containing CNF, it was found that the measured particle sizes could be determined both by the size of CNF and by those associated with polymer molecules. Thus, the central region with an average content of CNF and carrageenan represents larger particles. It should be noted that there may be some synergistic effect in which aggregation of CNF and/or polymers in this area may occur. The data for nanodispersions containing CNC reflect that the region with the highest content of CNC and the lowest content of carrageenan has smaller particle sizes. This may indicate that under these conditions, better dispersion of CNC occurs as well as better dispersion of polymers without aggregation of molecules and particles. While the hydrogen bonding between CNF still could lead to aggregation at the micro level.^28^

**Figure 5:**
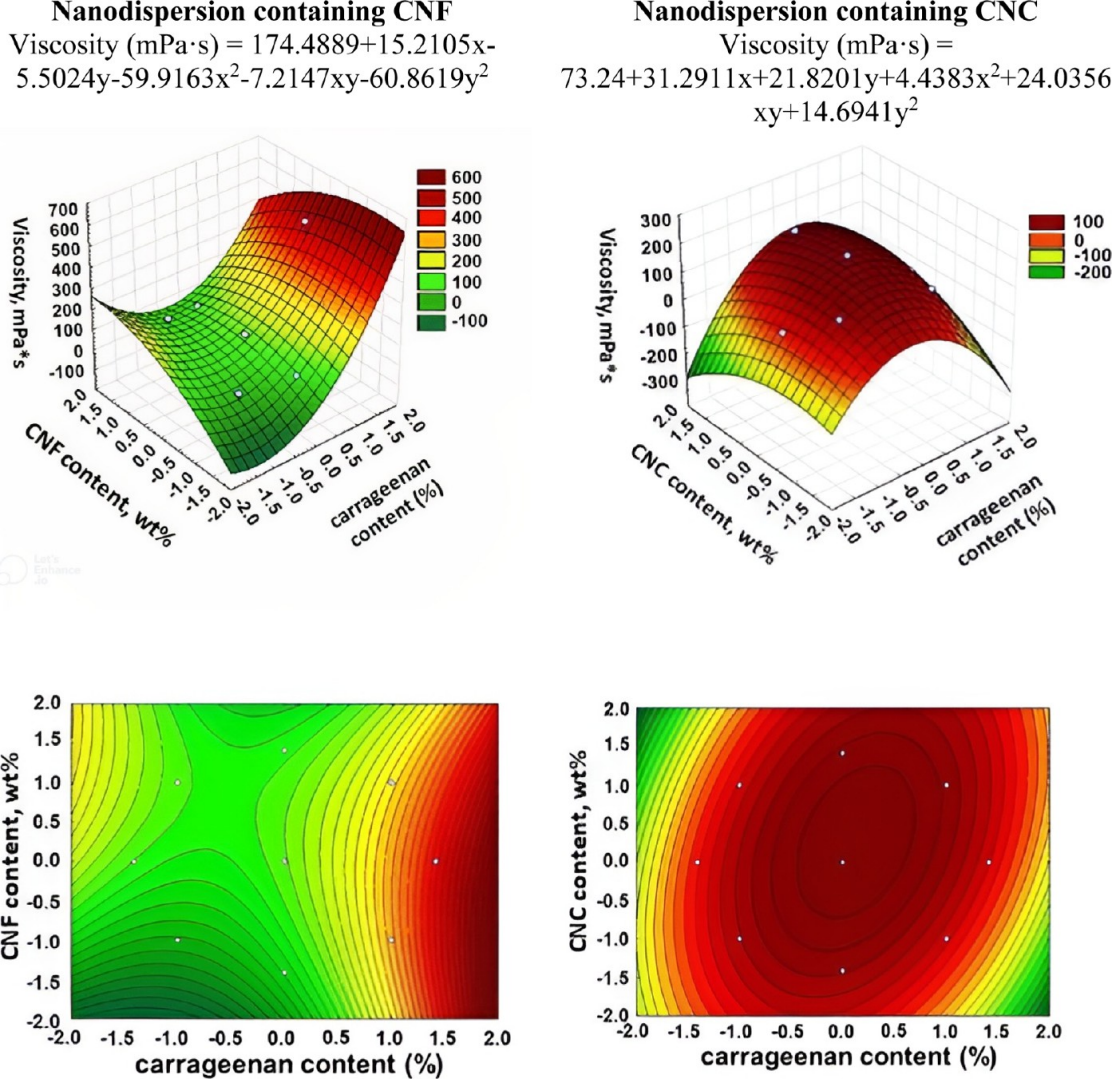
3D surface and contour plot graphs for the dependence of the kinematic viscosity of nanodispersions.

The most important properties of dispersions for deposition on products are viscosity and contact angle relative to the surface to which it is applied. Viscosity of the nanodispersions significantly affect the process of applying the liquid onto fruits/vegetables.^29^ If the liquid is very viscous, such an application will be difficult and require complicated equipment suitable for more viscous liquids, making the final application process more expensive. Despite this, solutions with higher viscosities (optimal) improve the adhesion of the liquid to the surface of the solid and also help in fixing components used in the formulation, ensuring better dispersion of these in the solution. The obtained results of kinematic viscosity data from the statistical treatments are presented in Figure 5.

**Figure 6:**
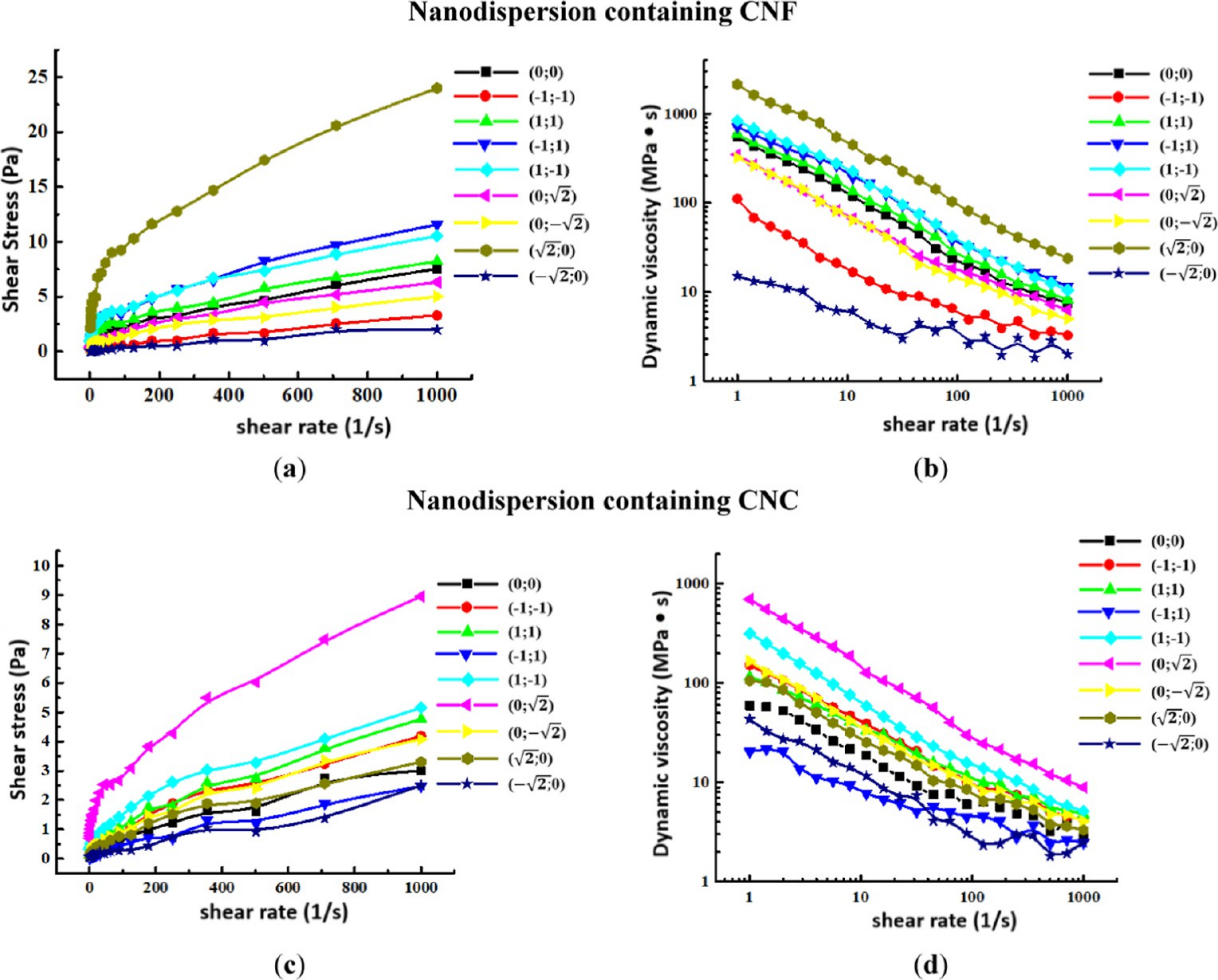
Dependence of shear stress on shear rate (a,c) and dynamic viscosity on shear rate (b,d) for nanodispersions containing CNF and CNC.

The formulations with a high carrageenan content (lower starch content), regardless of CNF content, produce solutions with greater viscosity. Therefore, the presence of carrageenan affects viscosity to a greater extent probably because it is a polymer with a greater molar mass than the starch used in this study.^30^ Therefore, the choice of optimal viscosity depends on the variation in the starch/carrageenan composition and not on the other components of the formulation. For nanodispersions with CNC, the highest viscosity values were obtained for the central region, where intermediate levels of NC and carrageenan were used. Despite this, the viscosity variation in the other regions was not very large, always below 100 mPa·s; these values being much lower than those observed for nanodispersions with CNF. The use of CNF results in solutions with much higher viscosity due to structural features as high aspect ratio and degree of entanglement.^31^Figure 6 presents the obtained data of shear stress vs shear rate and dynamic viscosity vs shear rate for nanodispersions.

**Figure 7:**
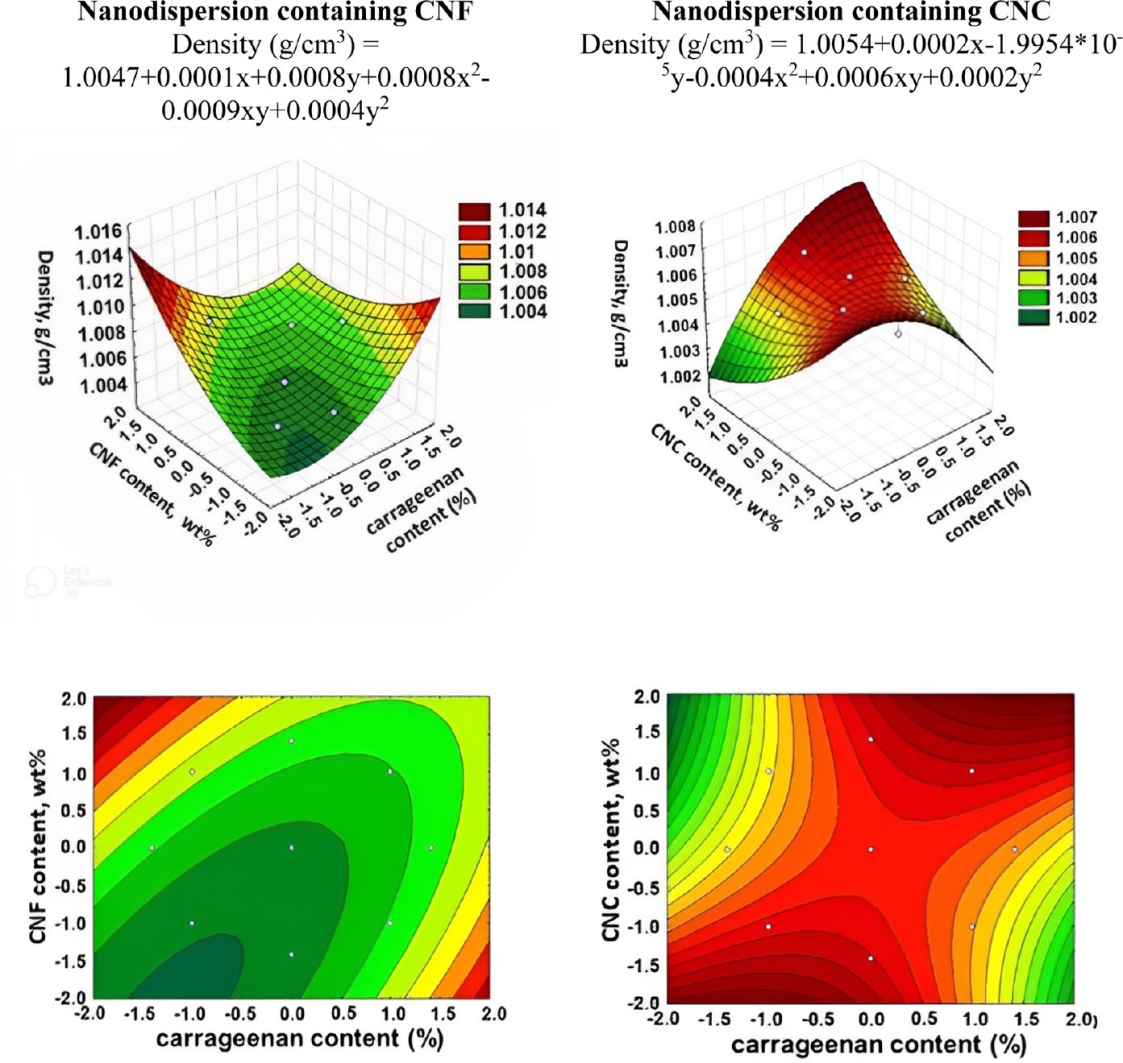
3D surface and contour plot graphs for the dependence of the density of nanodispersions.

The presented dependences demonstrate that all prepared dispersions belong to non-Newtonian liquids, which may be due to the fact that suspended particles are present in the fluid system.^32^ The plastic flow of these nanodispersions occurs because the particles, in contact with each other, form an internal frame. The flow of such a system occurs only after the destruction of its structure (framework), which determines (ensures) the mobility of particles relative to each other. If the dispersions were purely viscous Newtonian fluids, then after applying these dispersions to the surface of vegetables and fruits, after some time they would have to be drained from their surface. Consequently, the layer remaining on the surface indicates that the dispersion has the properties of a solid. A detailed examination of the dependence of shear stress on shear rate shows that the flow curve is nonlinear, namely, the dispersions are related to non-Newtonian pseudoplastic media and viscoplastic media due to the high yield strength.^33^ The density of nanodispersion samples is presented in Table 3 and Figure 7.

**Table 3:** Density of Nanodispersions

sample	nanodispersion with CNF		nanodispersion with CNC	
	density g/cm^3^	standard deviation g/cm^3^	density g/cm^3^	standard deviation g/cm^3^
(0;0)	1.00470	0.00010	1.00460	0.00010
(−1;–1)	1.00470	0.00003	1.00646	0.00009
(1;1)	1.00594	0.00002	1.00540	0.00010
(−1;1)	1.00916	0.00005	1.00461	0.00002
(1;–1)	1.00496	0.00003	1.00494	0.00003
(0; )	1.00550	0.00010	1.00601	0.00009
(0;)	1.00503	0.00004	1.00514	0.00003
(;0)	1.00740	0.00020	1.00518	0.00002
(;0)	1.00471	0.00007	1.00374	0.00002

Although the variation range of nanodispersion density values is very low, the lowest values tend to be obtained in formulations with lower carrageenan contents. This shows that formulations with a higher starch content produce solutions with lower densities than formulations with a higher content. However, starch has a higher density compared to carrageenan.^34^ It should be noted that nanodispersions with CNC have lower density values compared to ones with CNF due to structural features as high aspect ratio and degree of entanglement.^31,34^

**Figure 8:**
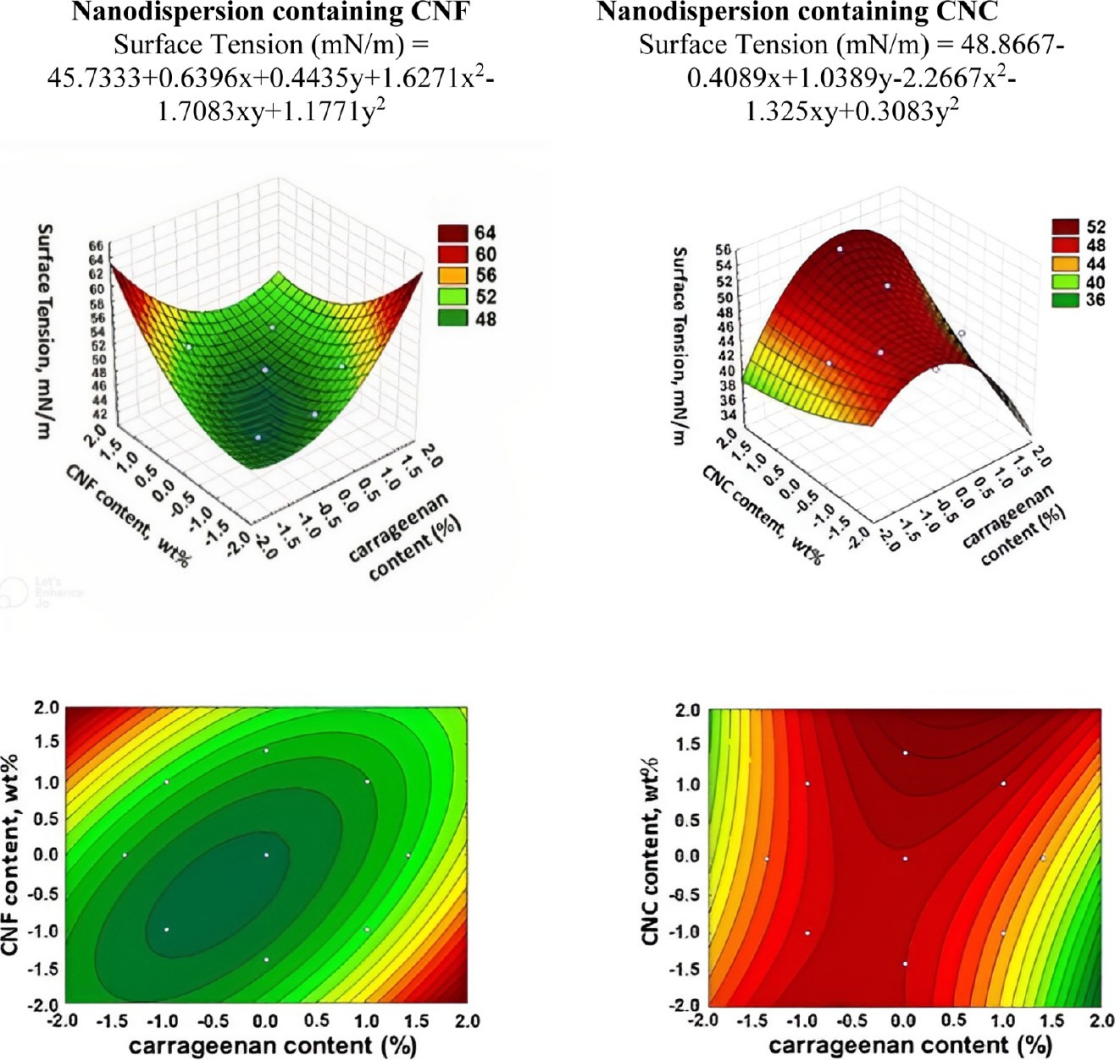
3D surface and contour plot graphs for the dependence of the surface tension of nanodispersions.

Knowing the surface tension values of the solutions, those samples of fruits/vegetables that exhibit lower contact angle values indicate that their surface energy values are closer to the surface tension values of the solution and hence have greater affinity and better spreading/adhesion. Those that have higher values of contact angle show the opposite; that is, they have values of surface energy that are more distant from those of the surface tension of the solution and thus repel each other. The surface tension of nanodispersions determines whether the liquid would spread over the surface of the solid and have good adhesion to the product. To be applied to products, the nanodispersions and solid surface of product (fruit/vegetable) must have similar solid surface energy and liquid surface tension, indicating that they will have a similar chemical character and affinity. The surface tension values of nanodispersions are presented in Figure 8.

For nanodispersions containing CNF, the lowest surface tension values were obtained for the region with a lower carrageenan content (higher starch content) and lower CNF content. Also, surface tension values are very high with a combination of high CNF content and low carrageenan content as well as high carrageenan content and low CNF content. The lowest surface tension values for nanodispersions containing CNC are observed both for the region with a higher carrageenan content and a lower CNC content and for the region with a higher CNC content and a lower carrageenan content. Thus, it can be said that for coating nonpolar solid surfaces with lower surface energy values, it would be preferable to use solutions from regions with lower surface tension values. And for more polar solid surfaces with higher surface energy values, it is necessary to use solutions with higher surface tension values. In general, the use of CNC in nanodispersion formulations results in solutions with lower surface tension values than those using CNF. Thus, the type of nanoparticles used influences the surface tension behavior. This means that for nonpolar solid surfaces or with lower surface energies, nanodispersions with CNC will be more suitable as this is likely to result in better adhesion and spreading.

**Figure 9:**
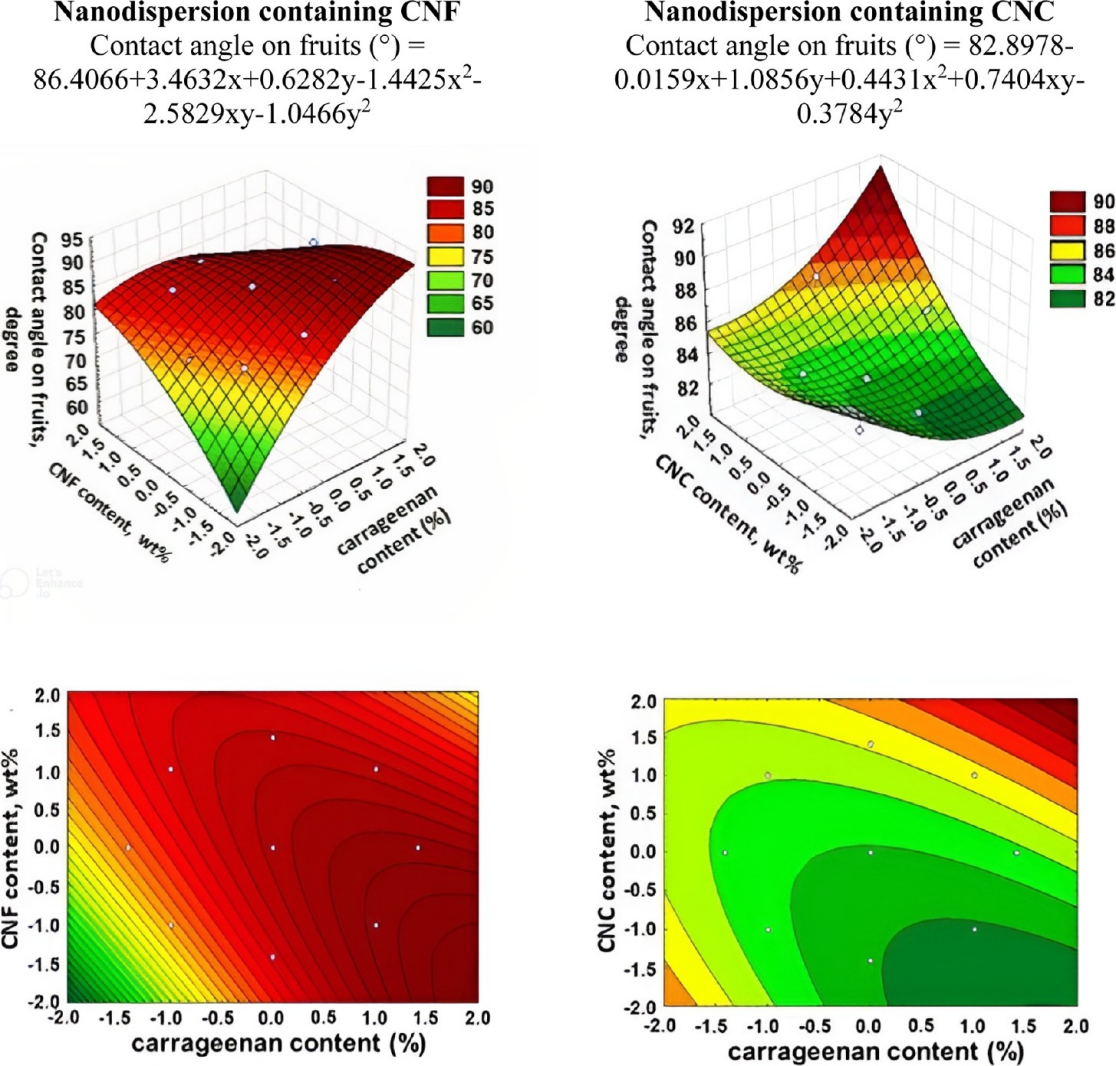
3D surface and contour plot graphs for the dependence of the contact angle of nanodispersions onto apples.

**Figure 10:**
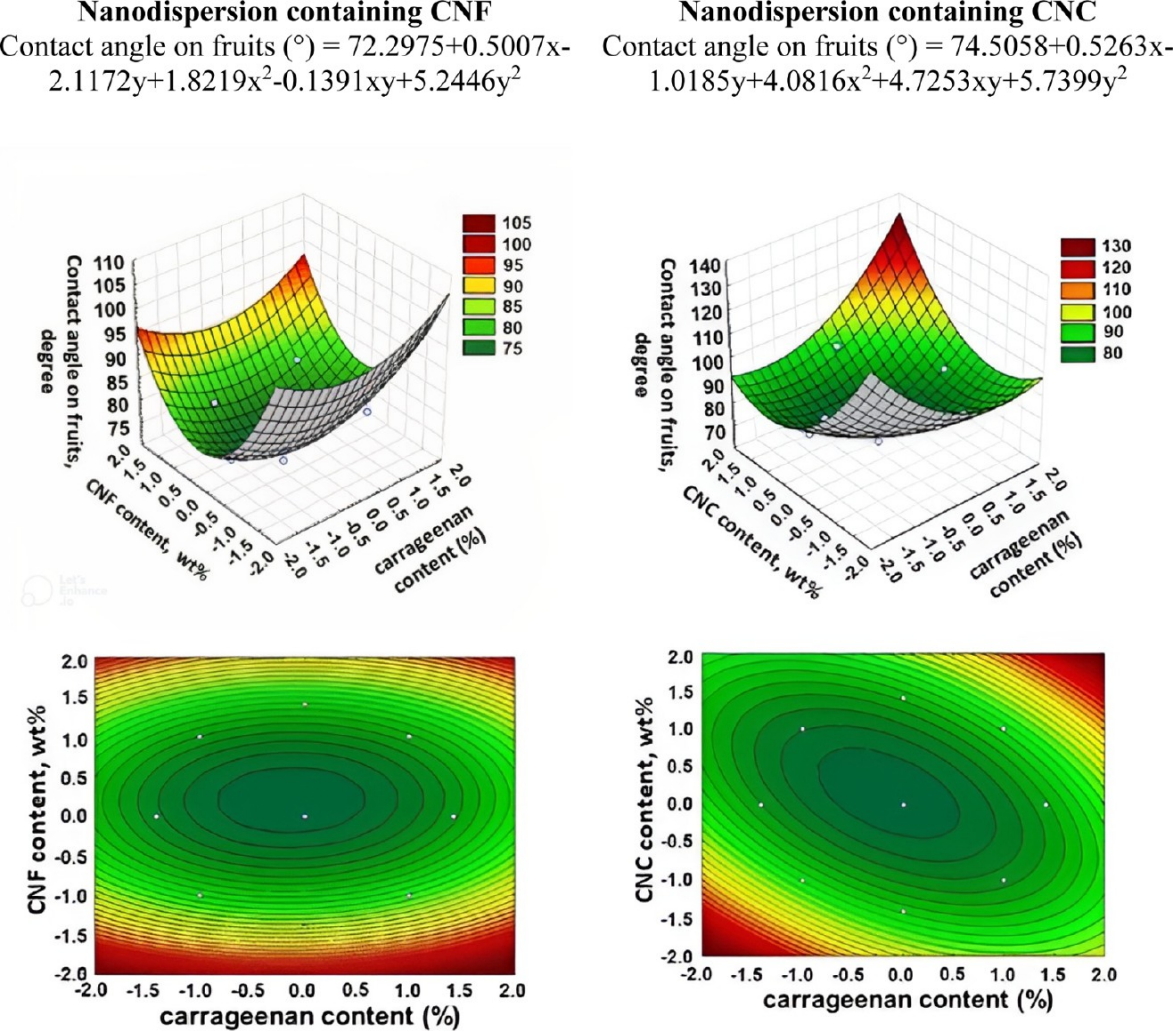
3D surface and contour plot graphs for the dependence of the contact angle of nanodispersions onto bananas.

**Figure 11:**
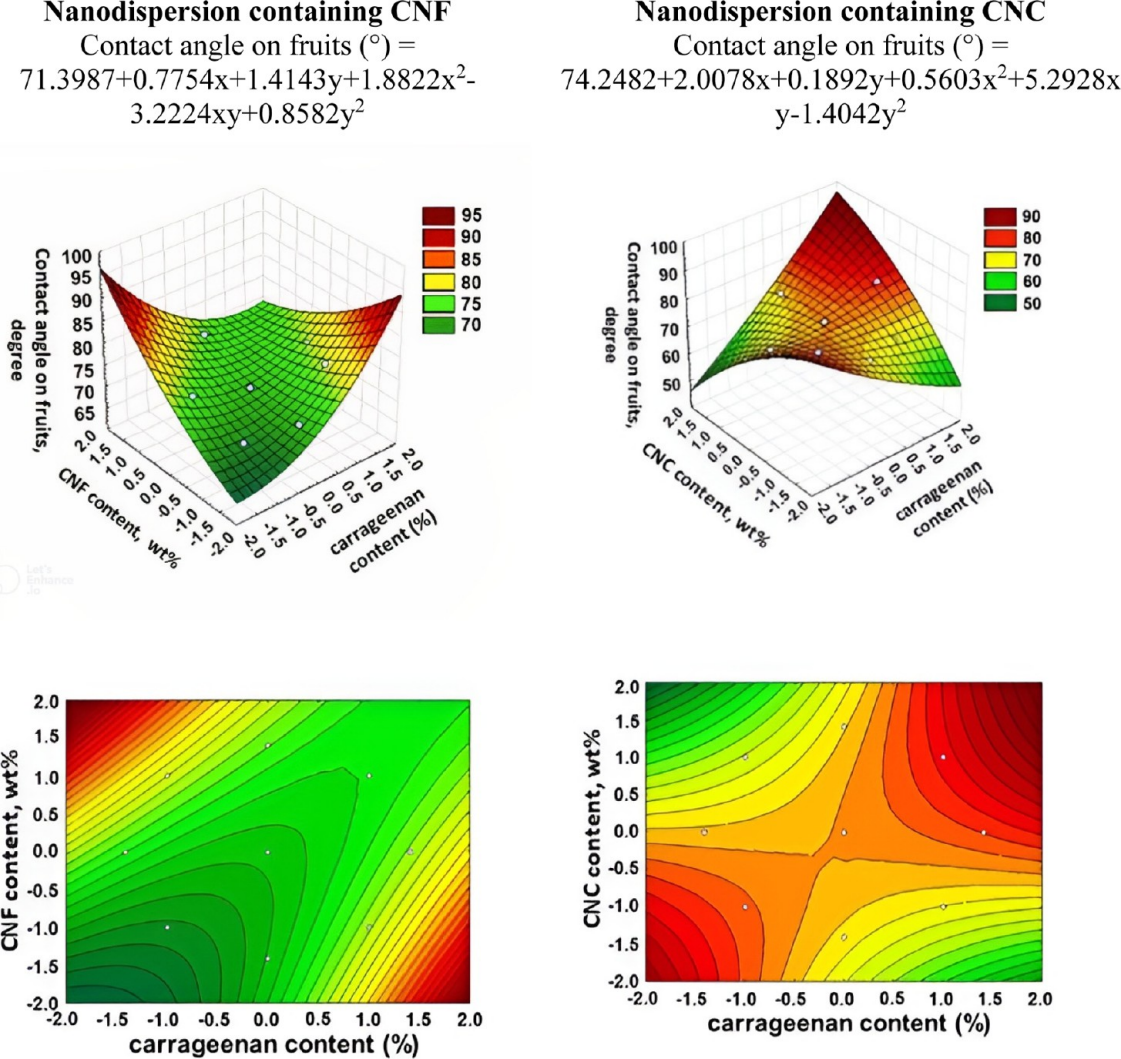
3D surface and contour plot graphs for the dependence of the contact angle of nanodispersions onto sweet peppers.

The nanodispersion surface tension values were around 45–50 mN/m, and to have good spreading and lower contact angles the used fruits/vegetables need to have surface energy values closer to these values. The surface polarity character of fruits/vegetables (namely, apple, banana, and sweet pepper) were evaluated through the contact angle measurements by the deposition of nanodispersions on them. The choice of these products was because they had different acidity or alkalinity (apple as an acid fruit (pH ∼ 3.5), banana (pH = 4.5–5.2) with mildly acidity, sweet pepper with low acidity (pH = 4.65–6.17)) with a short shelf life, and they are available and actively used by humans.^35^ Since the contact angle is directly related to the surface energy values of the solid and the surface tension of the dispersion,^36^ the contact angle measurements allow determining whether the nanodispersion spreads over the surface of the solid fruits/vegetables and whether it has good adhesion with the solid. When the contact angle value is more than 90°, there is no wetting of the solid by the liquid; that is, there is no spreading of the liquid on the surface of the solid. And when the contact angle value is less than 90°, there is wetting and the liquid spreads spontaneously.^37^ The contact angles of nanodispersions deposited onto fruits/vegetables (apple, banana, and sweet pepper) are presented in Figures 9–11.

It was demonstrated that the contact angle values obtained were less than 90°. The lowest contact angle values for apples (Figure 9) were found in the regions with the lowest CNF and carrageenan contents. The regions with high contact angle values for apples (close to and above 90°), i.e., higher carrageenan contents and lower CNF contents, are an unsuitable region for the nanodispersion formulation to be applied to apples due to low adhesion and spreading on the surface. The lowest contact angle values for apple for nanodispersions with CNC are located in the region containing higher carrageenan contents and lower NC contents. In this area with the lowest contact angle values, the best distribution of the nanodispersion over the apple surface occurs and, consequently, the best adhesion of the solution. This is coincident with the region of formulation of lower surface tension levels of solutions containing CNC. Thus, the more nonpolar apple surface with lower surface energy values is confirmed, and therefore, to obtain a good spreading of the nanodispersions on this fruit, it is necessary to use solutions with lower surface tension values (Figure 8). However, if we want to use solutions in this area (with a large contact angle value), there is still the possibility of adding surfactants to the formulation in order to reduce the surface tension of the solution and improve the distribution on the apple surface.^38−40^ However, in this case, it will be necessary to change the formulation by adding a new component.

The result obtained for sweet peppers (Figure 11) deposited with nanodispersions with CNF is similar to that observed for apples. The lowest contact angle values are for solutions with lower CNF and carrageenan contents. However, even with combinations of intermediate amounts of CNF and carrageenan, and also larger amounts of CNF and carrageenan, contact angle values much lower than 90° are obtained, which also shows that these combinations result in good spreading on the surface of the sweet peppers and, consequently, result in good adhesion of the solution to the surface. The lowest contact angle values for nanodispersions with CNC were obtained by the combination of opposite concentration values (lower carrageenan and higher NC contents and higher carrageenan and lower NC contents). In these regions, the spreading of solutions is favored due to the low contact angle values and results in better adhesion of the solutions to the surface. However, in general, the values obtained in other regions are below 90°, which also causes spreading and adhesion of the solution on the surface of the solid.

The behavior for bananas (Figure 10) differs from those observed for apples and peppers. In this case, the lowest contact angle values are observed for nanodispersions with intermediate CNF contents regardless of the carrageenan content. In other words, a wide range of formulations with different carrageenan compositions can be used but keeping the CNF levels close to the central point values is required to obtain good spreading and adhering the solution to the surface of the banana. For nanodispersions with CNC, contact angle values lower than 90° are located in the central region (combination of intermediate CNC and carrageenan contents). This is the optimal region for spreading and for better adhesion of the solution on the surface of this fruit. However, each fruit/vegetable has different compounds on the surface, changing their surface energies and may present different contact angle behavior with the prepared solutions.

It is also worth noting that the contact angle values of the nanodispersions decreases slightly in the order of apple > banana > sweet pepper. The pH of the fruit/vegetable surface can affect the contact angle measurements because it changes the ionic activity at the surface–liquid interface. This can lead to a change in the contact angle value. For fruits and vegetables with different acidities (apple as an acid fruit, banana with mildly acidity, sweet pepper with low acidity), this is more likely to be connected with the pulp (internal part) than the shell (external part) of samples. There may be other compounds on the outside, often waxes, that protect the fruit, and these compounds are usually neutral in nature. However, in this study, even if there was a compound on the surface that was ionized, it would be in small quantities and would have had very little effect on the contact angle values. In addition, whether there were ionizable compounds on the surface of the fruits and vegetables under study, the contact angle measurements were to check whether the nanodispersion would spread over the surface or not.

### 3.2 Nanodispersion Deposition on Fruits

**Figure 12:**
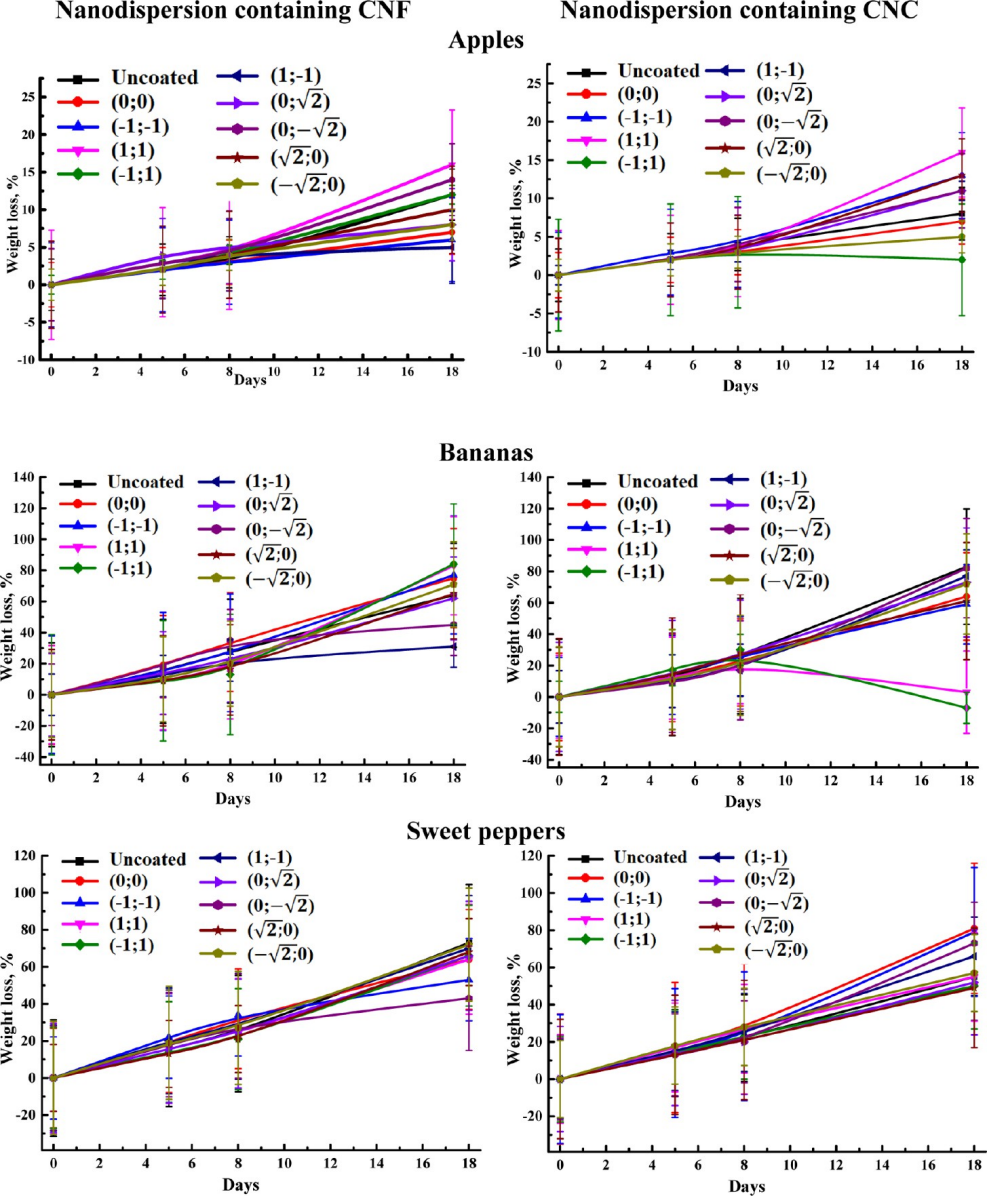
Weight loss of coated and uncoated fruits/vegetables during storage for 18 days. Vertical bars represent the standard deviation (*n* = 3).

Figure 12 shows the effect of a nanodispersion coating on the weight loss of apples, bananas, and sweet peppers during the storage.

Weight loss increased throughout the storage period for uncoated and coated with nanodispersion samples with significant differences. Uncoated apples, bananas, and sweet peppers had the weight loss of 8–12%, 64–83%, and 55–73%, respectively, after 18 days. The weight reduction of the samples with/without coating is in the order of apples < sweet peppers < bananas, which corresponds to the shelf life of these samples.^41−43^ The application of some nanodispersion formulations resulted in greater weight loss compared to other coated samples, which could be due to the uneven spreading and film formation on the solid surface because of the slight variation of density, viscosity, higher contact angle, and surface tension described earlier. The reduced values for banana mass loss for the samples coated with (1;–1) and (0;√2) nanodispersion with CNF and (1;1) and (−1;1) nanodispersion with CNC were due to the severe and rapid rotting of the bananas. Based on the obtained data and as described previously, it was demonstrated that the nanodispersion with CNF of (−1;–1) composition and the nanodispersion with CNC of (−1;1) composition led to decreased water loss of these fruits/vegetables. The advantage of developing the coating from these nanodispersions could be that this experiment was carried out under warm conditions (27 °C), while it has been frequently conducted at low temperatures.^44,45^

### 3.3 Investigation of Optimal Coatings from Nanodispersions

**Figure 13:**
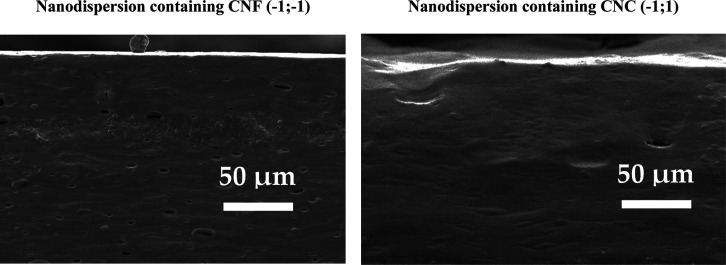
Cross-sectional SEM microphotographs of films containing CNF and CNC.

Additionally, films prepared from these nanodispersions ((−1;–1) with CNF and (−1;1) with CNC) were investigated by various methods such as scanning electron microscopy (SEM), climatic tests, optical density, light transmission, and water solubility measurements. SEM cross-sectional micrographs of these films are presented in Figure 13.

The SEM microphotographs demonstrate that the films have a homogeneous cross-sectional structure with a uniform distribution of NC throughout the matrix, showing no visible aggregation of particles. To prevent aggregation, NC was utilized in the form of an aqueous dispersion, which helped to minimize its agglomeration within the polymer matrix.^46^ The presence of some holes is attributed to air bubbles trapped during the film preparation process. Overall, the mixture of starch/carrageenan/NC was compatible, resulting in films with visually homogeneous cross-sectional structures without any phase separations. The physicochemical and electrostatic interactions between carrageenan and starch, taking into account the intrinsic characteristics of each component, have already been widely discussed in the literature.^47−50^ It was confirmed that the blending of carrageenan and starch led to the formation of a strong polymer matrix and generation of stronger intermolecular tensile strength.^51^ An earlier study also showed that the introduction of NC into this blend matrix resulted in the formation of a hydrogen bonding system between polysaccharides.^8^ However, it has previously been shown that varying the composition of the starch/carrageenan/NC mixture can change the characteristics of the resulting material, which allows it to be optimized for a specific task and specific application to a particular fruit/vegetable.

The films prepared from these nanodispersions ((−1;–1) with CNF and (−1;1) with CNC) were tested during climatic tests at temperatures from −60 to 60 °C for 20 cycles. No defects, cracks, or changes in geometric parameters were detected on any of these samples. All of these films made from nanodispersions successfully passed climatic tests, which corresponds to a guaranteed shelf life of 5 years and stability at low storage temperatures.

**Figure 14:**
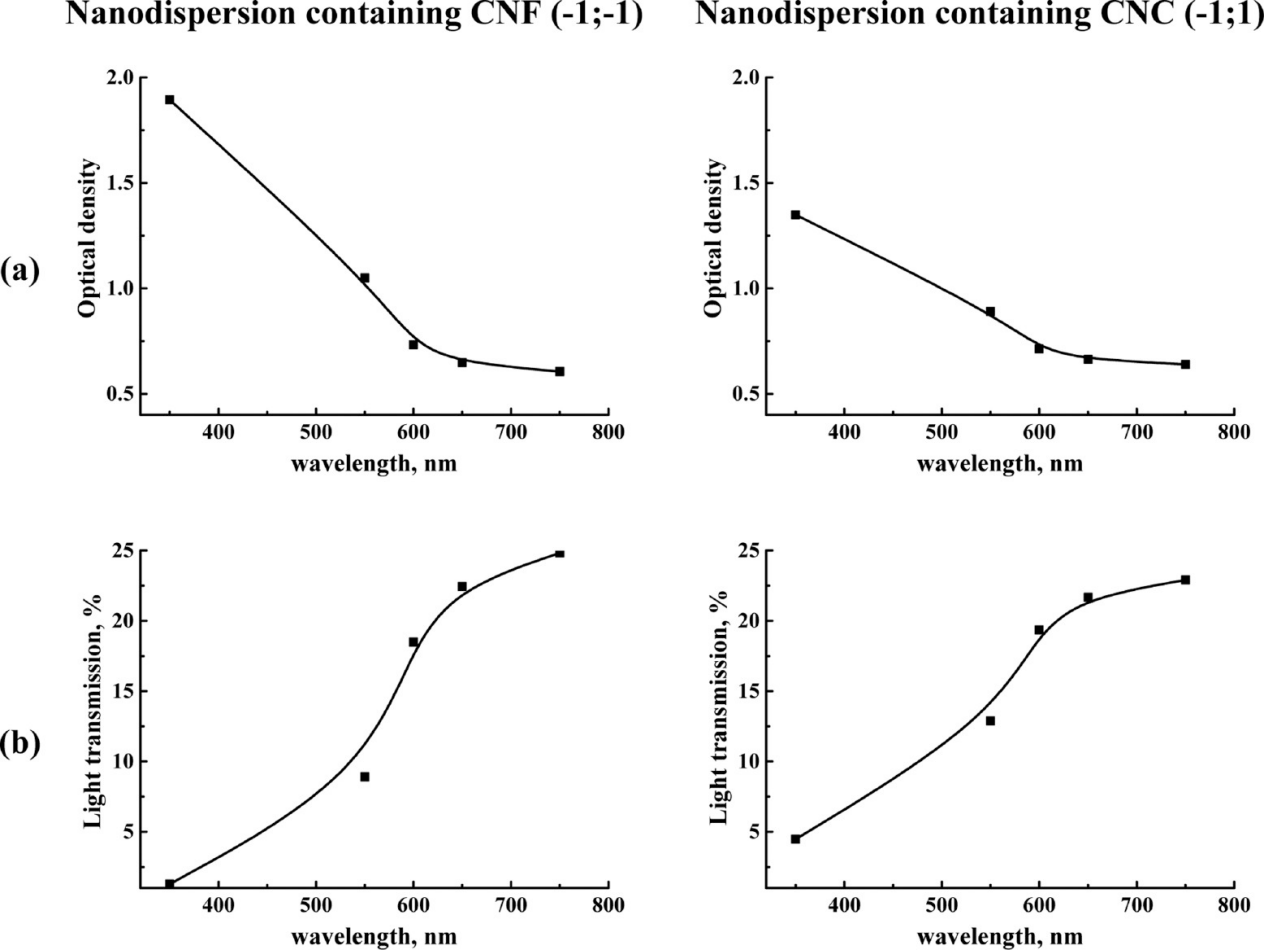
(a) Optical density and (b) light transmission properties of films prepared from nanodispersions containing CNF and CNC.

To evaluate the transparency of the coating material, the optical density and light transmission of the prepared films from nanodispersions with optimal compositions ((−1;–1) with CNF and (−1;1) with CNC) were measured at 350–750 nm wavelengths. The selected wavelength range was chosen to examine the response to visible and UV radiation. The data obtained are shown in Figure 14.

The films displayed the highest optical densities at 350 nm, effectively blocking radiation at this wavelength, indicating their strong UV radiation barrier properties. Between 550 and 600 nm, the films had lower optical densities, permitting some radiation to pass. At 650 and 750 nm, the optical densities were minimal. The films exhibited low light transmittance in the UV range (up to 400 nm), but as the wavelength increased in the visible range (550–750 nm), the light transmittance rose to 30%. It is crucial for food packaging that the deposited coating allow some light to pass for product visibility. The tested films from nanodispersions fulfill this requirement, demonstrating adequate transmittance in the visible range.

These data confirmed that coating from nanodispersions may act as a barrier in the UV range, helping to minimize oxidation in packaged food products. This may indicate that these developed nanodispersions can be used immediately by spraying on fruits on trees/bushes during harvesting, and it will be stable to UV light and high temperatures and will not harm the environment.^8^ It is also worth noting that when studying films made of nanodispersions with optimal properties ((−1;–1) with CNF and (−1;1) with CNC) for dissolution in water, a 54% value was obtained in an hour. It indicates that the coating from these nanodispersions can be washed off from fruits/vegetables only with prolonged contact with water, but this is not necessary since it is edible.

## 4 Conclusions

In this study, a sustainable edible coating in the form of a nanodispersion from carrageenan/starch/NC was developed and studied to enhance fruit/vegetable shelf life and preservation. To determine the optimal nanodispersion formulation, various compositions with different polysaccharide ratios and types of NC particles (CNF and CNC) were evaluated by analysis of particle shape, size and distribution, viscosity, density, surface tension, and contact angle on apples, bananas, and peppers and by evaluation of storage after nanodispersion coating. It was shown that the most promising nanodispersions for coating application were those with a low content of carrageenan (33.3%) and up to 5% CNF and 10% CNC. This difference in the NC concentration was due to the structure and size of its particles. This developed nanodispersion can be used prospectively by spraying on fruits/vegetables during harvesting, and it will be resistant to UV radiation (confirmed by optical density and light transmittance measurements) and high temperatures and will not harm the environment and will be edible since it consists of components already actively used in the food industry.
